# Clinical study on postoperative recurrence in patients with pN0 esophageal squamous cell carcinoma

**DOI:** 10.1186/s13019-014-0150-4

**Published:** 2014-08-28

**Authors:** Xu-feng Guo, Teng Mao, Zhi-tao Gu, Chun-yu Ji, Wen-tao Fang, Wen-hu Chen

**Affiliations:** Department of Thoracic Surgery, Shanghai Chest Hospital, School of Medicine, Shanghai Jiaotong University, 241 West Huaihai Road, Shanghai, 200030 China

**Keywords:** Esophagus neoplasms, Lymph node dissection, Recurrence and metastasis

## Abstract

**Background:**

Despite increasingly radical surgery for esophageal carcinoma, many patients still develop tumor recurrence after operation. This study was designed to analyze the clinical and pathologic influencing factors of early recurrence in patients with histological node-negative (pN0 stage) esophageal squamous cell carcinoma (ESCC) after radical esophagectomy.

**Methods:**

A retrospective study on 112 consecutive pN0 stage ESCC patients who underwent esophagectomy with lymphadenectomy by the same surgical team from January 2004 to December 2010. There were 92 male and 20 female patients, aging from 36 to 80 years with a mean age of 60.3 years. The Cox proportional hazards model was used to determine the independent risk factors for recurrence within 3 years after the operation.

**Results:**

Recurrence was recognized in 45 patients (40.2%) within 3 years after operation. The median time to tumor recurrence was 17.4 months. Locoregional recurrence was found in 38 patients (33.9%) and hematogenous metastasis in 7 patients (6.3%). However, locoregional recurrence accounted for 84.4% of all relapse patients. Recurrence closely correlated with tumor location, grade of differentiation, primary tumor stage (pT) and pathologic stage (χ^2^ = 6.380 to 18.837, *p* < 0.05). The Cox multivariate analysis showed that upper/middle thoracic location (OR = 1.092, *p* = 0.049) and pT3-4a stage (OR = 3.296, *p* = 0.017) were independent risk factors for postoperative locoregional recurrence.

**Conclusion:**

Locoregional recurrence was the most common recurrence pattern of patients with pN0 ESCC within 3 years after operation. Upper/middle thoracic location and pT3-4a stage were independent risk factors for locoregional recurrence of pN0 ESCC after radical esophagectomy.

**Electronic supplementary material:**

The online version of this article (doi:10.1186/s13019-014-0150-4) contains supplementary material, which is available to authorized users.

## Background

In China, the predominant pathologic type of esophageal carcinoma is squamous cell carcinoma (ESCC), and many tumors locate in the middle thoracic. The overall 5-year survival rate of patients with lymph node metastasis (LNM) after surgical resection was only about 20% [1], and most of these patients died of tumor relapse. Recently, the therapeutic efficacy for ESCC had been greatly improved due to the establishment and promotion of multidisciplinary treatment model [2]. However, even for histological node-negative (pN0 stage) ESCC patients, a large number of these patients still experienced recurrence after operation [3]. So it was important to study the causes and characteristics of recurrence when designing therapeutic strategies, including operative procedures and combined therapies.

The purposes of this study were to explore the pattern and time of recurrence after extended radical esophagectomy with systemic lymphadenectomy, to identify the prognostic factors responsible for recurrence, and to discuss therapeutic strategies that might be helpful to decrease recurrence and improve survival.

## Methods

### Patients

This study was approved by the Research Ethics Committee of Shanghai Jiao Tong University School of Medicine and all participants at the Shanghai Chest Hospital gave written informed consent. A total of 112 consecutive pN0 stage ESCC patients who underwent esophagectomy with lymphadenectomy by the same surgical team from January 2004 to December 2010. The inclusion criteria were as follows: (1) routine preoperative esophageal endoscopy, a clear pathology of squamous cell carcinoma, (2) assessment of tumor location by the upper gastrointestinal barium swallow, (3) no evidence of tumescent cervical or supraclavicular lymph node disease was noted on physical examination, and a preoperative computed tomograpy (CT) scan or cervical B ultrasound study indicated no cervical or supraclavicular lymph node metastasis, (4) positron emission tomography to rule out distant metastasis, (5) no preoperative radiotherapy and/or chemotherapy, (6) pathologically confirmed lymph node-negative after operation, (7) all the patients were R0 resection of pathologically confirmed, (8) all the patients did not receive any adjuvant therapy before recurrence. Of all the patients, there were 92 men and 20 women, and the median age of 60.3 years (range: 36–80 years). The tumor location and the TNM classification were determined according to criteria established by the Union for International Cancer Control (UICC) in 2009 [4].

### Surgical procedure

At operation the patient was placed in the 90° left lateral decubitus position. After a right posterolateral thoracotomy, the chest was entered through the fifth intercostal space. The azygos vein arch was divided, and the esophagus was dissected from the esophagogastric junction to the apex of the chest. When the tumor invasion obviously extended outside the esophagus, the thoracic duct was routinely ligated above the diaphragm. An upper midline abdominal incision was also made, and the abdomen was explored. During mobilization of the stomach, care was taken to preserve the right gastroepiploic vessels and arcades. The left gastric artery and vein were isolated and doubly ligated at their origin. The hiatus was enlarged and the stomach was pulled into the chest. An end-to-side esophagogastric anastomosis was performed within the apex of the chest (above the azygos vein) and the stomach was secured into the mediastinum (Ivor-Lewis).When the tumor located in upper thoracic, we will pull the stomach to neck by post-sternum tunnel for anastomosis (McKeown).

**Figure 1:**
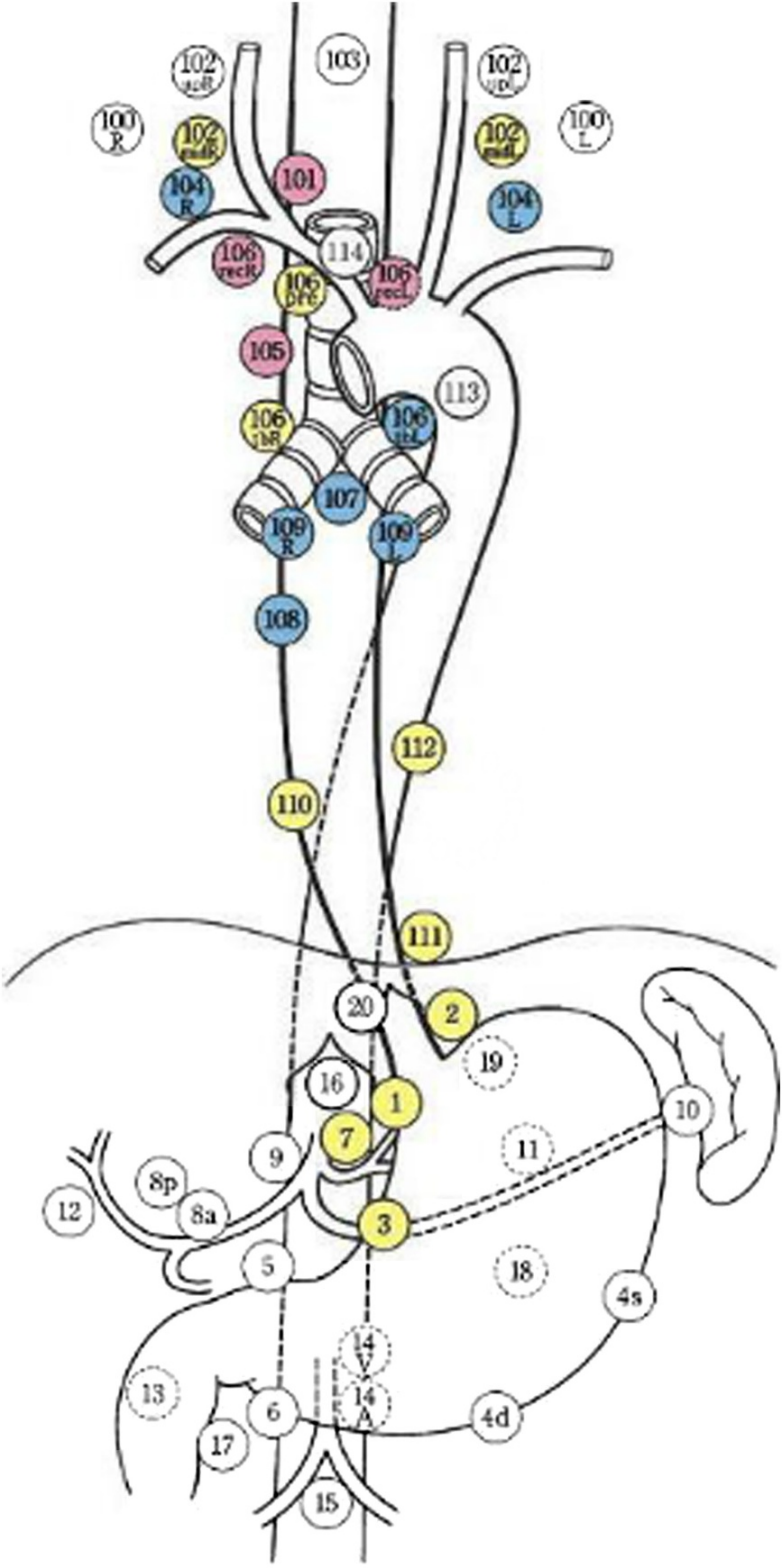
**Regional lymph node stations for staging esophageal cancer (JEOG).**

According to a lymph node mapping system for esophageal cancer (Japanese esophageal oncology group, JEOG) (Figure 1), thoraco-abdominal 2-field lymphadenectomy was undertaken. Some patients were undertaken cervico-thoraco-abdominal 3-field lymphadenectomy based on the positive results of preoperative cervical ultrasonography. The fields of lymph node dissections were as follows: 100, superficial cervical lymph nodes; 101, cervical esophageal lymph nodes; 102, deep cervical lymph nodes; 104, Supraclavicular lymph nodes; 105, upper thoracic esophageal lymph nodes; 106, recurrent laryngeal nerve lymph nodes; 107, subcarinal lymph nodes; 108, middle thoracic esophageal lymph nodes; 109, hilar lymph nodes; 110, lower thoracic esophageal lymph nodes; 111, diaphragmatic lymph nodes; 1, cardial lymph nodes (right); 2, cardial lymph nodes (left); 3, lesser curvature lymph nodes; 7, left gastric lymph nodes.

### Follow-up examinations

Patients were routinely accepted the first examination at 3–4 weeks after the operation, examined at 3-month intervals for 1 year and at 6-month intervals thereafter. During each follow-up visit, the patient underwent a clinical evaluation, blood biochemistry examination including tumor markers, and chest radiography. Endoscopy, ultrasonography (US) of the neck and abdomen, and computed tomography (CT) of the neck, thorax, and abdomen were performed at least once a year. More selective investigations such as positron emission tomography (PET), bone scintigraphy, and Head-enhanced magnetic resonance imaging (MRI) were carried out based on specific symptomatology, clinical examination, and biochemical profile. Detection of a suspected recurrence at any one site was followed by a thorough detailed investigation to confirm or refute the occurrence and to examine every other site.

### Definition of locoregional and hematogenous recurrence

The first recurrence was noted, and any additional recurrence found within one month was considered to have occurred simultaneously. These lesions were classified as locoregional (at the remaining esophagus, the anastomotic site, or the mediastinum, cervical, supraclavicular and celiac axis lymph nodes) and hematogenous (in the distant organs such as liver, lung, bone and pleura, peritoneum) recurrence. Simultaneous locoregional and hematogenous recurrence was classified as a hematogenous recurrence.

### Statistical analysis

The SPSS 16.0 software package was used for data analysis. Data were expressed as median with ranges (minimum-maximum) or as percentages. The chi-square test was used to evaluate differences in clinicopathologic features. The Cox proportional hazards model was used to determine the independent risk factors for recurrence within 3 years after the operation. Estimation of recurrence was calculated with the Kaplan-Meier method, and the statistical differences were analyzed with the log-rank test. A *p* value of < 0.05 was considered statistically significant for all procedures.

## Results

112 patients were investigated and recurrence was recognized in 45 patients (40.2%) in the first 3 years after operation. The median disease-free interval until recurrence was 17.4 months (range: 3.5-26.8 months). Of these, 39 were men and 6 were women, and the median age was 56 years (range: 39–76 years).

### Pattern of recurrence

38 patients (33.9%) developed a locoregional recurrence; 7 patients (6.3%) developed a hematogenous recurrence, including 2 patients (1.8%) with simultaneous locoregional and hematogenous recurrence. However, locoregional recurrence accounted for 84.4% of all relapse patients. The relationship between clinicopathologic factors and recurrent disease was depicted in Table 1. There were significant differences in tumor location (*p* = 0.038), grade of differentiation (*p* = 0.043), primary tumor stage (pT) (*p* = 0.006) and pathologic stage (*p* = 0.024) between patients with recurrence and those without recurrence.

**Table 1:** **Clinical data of 112 pN0 ESCC patients undergoing radical esophagectomy with lymphadenectomy**

Variables	Recurrence		Statistic	***p***Value
	Positive	Negtive		
No. of patients	45	67	-	-
Gender (M/F)	39/6	53/14	*χ* ^2^ = 0.497	0.173
Age (years)	60 ± 5	61 ± 7	*t* = 4.877	0.782
Tumor location			*χ* ^2^ = 7.845	0.038
&Upper	11	10		
&Middle	29	35		
&Lower	5	22		
Grade of differentiation			*χ* ^2^ = 6.380	0.043
&Well (G1)	4	12		
&Moderately (G2)	9	22		
&Poorly (G3)	32	33		
**pT status**			*χ* ^2^ = 18.837	0.006
&T1	0	19		
&T2	6	19		
&T3	35	26		
&T4a	4	3		
**Pathological staging**			*χ* ^2^ = 7.845	0.024
&Stage I	1	20		
&Stage II	9	18		
&Stage III	31	26		
&Stage IIIA	4	3		
Lymphadenectomy			*χ* ^2^ = 5.226	0.119
&Two field	39	58		
&Three field	6	9		
Intralmural metastasis			*χ* ^2^ = 3.593	0.336
&Positive	4	0		
&Negtive	41	67		

### The distribution of the sites of tumor recurrence

**Figure 2:**
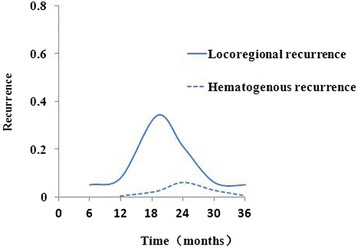
**The comparison of occurrence time between locoregional recurrence and hematogenous recurrence in pN0 ESCC patients.**

With respect to the locoregional tumor recurrence, 21 patients (15.3%) had recurrence in only cervical/supraclavicular lymph nodes, 13 patients (34.2%) had recurrence in only mediastinal lymph nodes, 6 (15.8%) in only abdominal lymph nodes. In cases of hematogenous recurrence, the main sites of recurrence were liver (6/7), lung (2/7), and bone (1/7). There was no one had recurrence in brain, pleura and omentum in our patients within 3 years after operation. Lymph node metastasis occured earlier than hematogenous recurrence (Table 2 and Figure 2).

**Table 2:** **Site of locoregional and hematogenous recurrence in 45 patients**

Site of recurrence	No.	Percent (%)	Time of recurrence^3^(month)
Locoregional recurrence^1^	38		
Cervical/supraclavicular node	21	55.3	14.9 (3.5 ~ 17.7)
Mediastinal node	13	34.2	
upper	9	23.7	17.2 (4.8 ~ 23.2)
middle	3	7.9	19.2 (7.1 ~ 25.3)
lower	1	2.6	23.1
Abdominal node	6	15.8	18.4 (4.5 ~ 26.8)
Anastomotic	1	2.6	8.6
Hematogenous recurrence^2^	7		
Liver	6	85.7	10.4 (5.2 ~ 20.4)
Lung	2	28.6	12.8 (9.4 ~ 16.2)
Bone	1	14.3	18.5
Others(brain/pleura/omentum)	0		

^1^Three patients experienced locoregional recurrence at more than one site.

^2^Two patients experienced hematogenous recurrence at more than one site.

^3^Mean (range).

### The multivariate analysis for locoregional recurrence

The multivariate analysis by the Cox proportional hazards model demonstrated that Upper/middle thoracic location (*p* = 0.049, OR = 1.092) and pT3-4a stage (*p* = 0.017, OR = 3.296) were significant risk factors for locoregional recurrence of pN0 ESCC after operation (Table 3). We did not find a significant correlation between locoregional recurrence and gender, histological differentiation, or lymphadenectomy style.

**Table 3:** **The multivariate analysis for locoregional recurrence of pN0 ESCC**

Variable	B	SE	Wald	***p***Value	OR	95 % CI
Tumor location						
(upper/middle versus lower)	1.087	0.557	3.808	0.049	1.092	1.088 ~ 2.383
Grade of differentiation						
(G3 versus G1 + 2)	0.478	0.235	4.143	0.068	1.613	0.690 ~ 1.551
Depth of invasion						
(pT3-4a versus pT1-2)	1.193	0.250	22.731	0.017	3.296	2.019 ~ 5.381

B: regression coefficient; SE: standard error; Wald: wald value; OR: odds ratio; CI: confidence interval.

**Figure 3:**
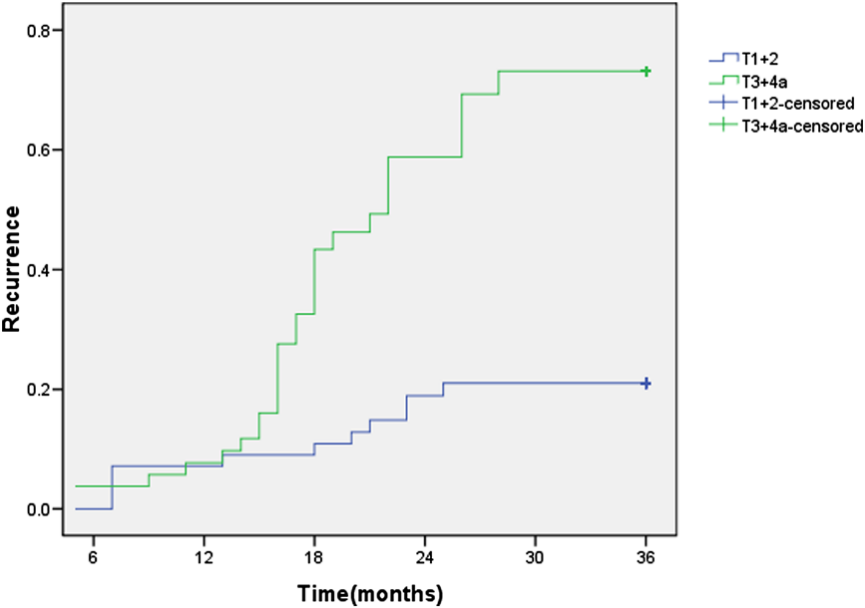
**Recurrence curve for the patients with different pT stage.**

**Figure 4:**
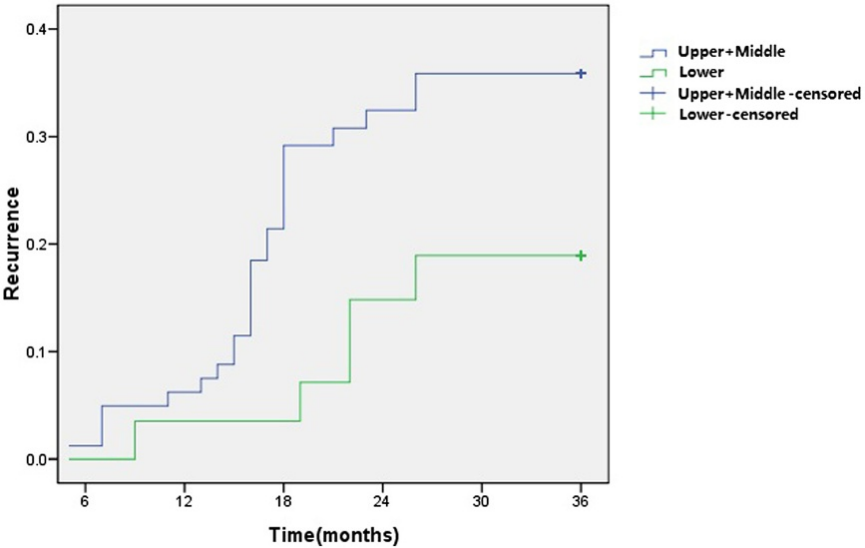
**Recurrence curve for the patients with different tumor location.**

The recurrence curve in pN0 patients with different pT stage was depicted in Figure 3 The log-rank test showed that there was a significant difference among the recurrence rates of these two groups (*p* = 0.000). The recurrence rate of the patients with upper/middle thoracic location was higher than the rate of lower thoracic location (*p* = 0.045) (Figure 4).

## Discussion

The 7th edition UICC TNM staging of esophageal cancer (2009) increased cell differentiation (G) and histological type (H) as a staging reference factor, also the pT3 stage (tumor invades adventitia) and pT4a stage (resectable tumor invading pleura, pericardium, or diaphragm) were distinguished [4]. With the wide application of the new version of the standard staging for esophageal cancer, the latest esophageal cancer treatment guidelines by National Comprehensive Cancer Network (NCCN) recommended for pN0 ESCC patients [5]: (1) pT1-4aN0M0 patients with ESCC, follow-up and observation after operation; (2) pT1-2N0M0 patients with adenocarcinoma, recommended follow-up and observation. However, some of the pT2N0M0 patients with high risk (poorly differentiated, lymphatic invasion, perineural invasion and <50 years of age), recommended postoperative chemoradiation; (3) pT3-4aN0M0 patients with adenocarcinoma were recommended postoperative chemoradiation or follow-up.

As we can see, prophylactic postoperative adjuvant therapy was not recommended for pT1-2N0M0 patients. However, there was no uniform international standards on whether pT3-4aN0M0 patients need postoperative adjuvant therapy or not, and what specific treatment should be chosen currently. It is worth noting that China, as a country with high incidence of esophageal cancer, nearly all of patients are squamous cell carcinoma. It is obviously different with the western countries in which the main histology of esophageal cancer was adenocarcinoma. Meanwhile, ESCC occur mostly in upper and middle thoracic, but adenocarcinoma occur mostly in lower thoracic. Whether from the perspective of anatomical or histological analysis, ESCC is more easy to have mediastinal lymph node metastasis even the neck and peritoneal metastasis than adenocarcinoma. Seen in this light, the foreign experience might not be suitable for China. Therefore, to study the risk factors for recurrence and metastasis in pN0 patients with ESCC, following suitable adjuvant treatment had important clinical significance for reducing recurrence and improving long-term survival.

Our cases showed that there was 40.2% of 112 pN0 ESCC patients had recurrence within 3 years after operation, with a median time (17.4 months) to recurrence, similar to the results reported in the literature [6]. Univariate analysis showed that recurrence closely correlated with tumor location, grade of differentiation, primary tumor stage (pT) and pathologic stage. The COX proportional hazards regression multivariate analysis confirmed that upper/middle thoracic location and pT3-4a stage were independent risk factors for postoperative locoregional recurrence. The pT stage (Wald = 22.731; OR = 3.296) was the most important risk factor on recurrence and metastasis of pN0 ESCC according to the Wald value. With increasing pT stage, the chance of recurrence and metastasis in pN0 esophageal cancer patients also increased significantly. The reason was that esophagus lacks serosa, once the tumor invades adventitia, not only the submucosal longitudinal lymphatic vessels but also lateral mediastinal lymphatic vessels originating from the muscle layer contribute greatly to the LNM [7]. In present study, the poorly differentiation (G3) did not have statistical significance with tumor recurrence might be due to small number of cases, but the *p* value (0.068) was close to 0.05. In addition, four patients with pathologically confirmed the presence of intralmural metastasis, three of them had cervical lymph node recurrence 2 years after operation and another appeared residual esophageal tumor recurrence 1 year after operation. It was suggesting that intralmural metastasis might be an important risk factor for recurrence of pN0 ESCC. Therefore, taking into account the possibility of multi-center origin in esophageal cancer and intralmural metastasis, we have to do the following in operation: (1) resection with sufficient length of esophagus; (2) complete resection of the lesion and the surrounding involved structures; (3) radical elimination of lymphatic tissue.

In the present study, the most common recurrence pattern of pN0 patients with ESCC after operation was locoregional recurrence (38/45, 84.4%) and LNM (37/38, 97.4%) was the main form, mainly in the cervicothoracic junction. Therefore, the cervicothoracic junction was the key position for preventing recurrence of pN0 patients with ESCC after operation. In addition, the difference of recurrence between two-field (thoracic and abdominal) lymph node dissections and three-field (cervical, thoracic and abdominal) lymph node dissections was not significant in our study. However, the literature had reported that three-field lymph node dissections could reduce recurrence and metastasis rate significantly [8]. The possible explanations were: Firstly, patients with pN0 ESCC might not need the three-field lymph node dissections; Secondly, there were statistical deviation due to small number of patients were given three-field lymph node dissections in this group, and this study was a retrospective analysis. Cervical B ultrasound was helpful to the diagnosis of LNM in cervicothoracic junction [9], combined with preoperative CT scan of chest/abdomen and positron emission tomography, establishing an accurate clinical staging was helpful to select the patients who do need three-field lymph node dissections [10]. We also took into account: (1) there were many important organs, large vessels in cervicothoracic junction and the region of recurrent laryngeal nerve was a relatively difficult position for systemic lymph node dissections because of higher requirements of surgical techniques; (2) three-field lymph node dissections had to face the bottleneck which can not further improve the long-term survival obviously. So, the postoperative radiotherapy and/or chemotherapy might be a good alternative.

Our data did not find independent risk factors for pN0 ESCC patients with hematogenous metastasis, suggesting that hematogenous metastasis was mostly influenced by the biological behavior of tumor cell rather than depth of tumor invasion, LNM and differentiation [11]. Thus, it was necessary to carry out prospective clinical study for evaluating whether vascular invasion, lymphatic invasion, and perineural invasion and so on were related with hematogenous metastasis or not.

## Conclusions

In summary, we have confirmed that locoregional recurrence was the most common recurrence pattern of patients with pN0 ESCC within 3 years after operation. Upper/middle thoracic location and pT3-4a stage were independent risk factors for locoregional recurrence of pN0 ESCC after radical esophagectomy. For these high-risk groups, prophylactic radiotherapy for bilateral supraclavicular and upper mediastinal might be helpful to reduce the recurrence rate.

## Authors’ original submitted files for images

Below are the links to the authors’ original submitted files for images. Authors’ original file for figure 1 Authors’ original file for figure 2 Authors’ original file for figure 3 Authors’ original file for figure 4

## Abbreviations

pN0 stage: Histological node-negative
ESCC: Esophageal squamous cell carcinoma
LNM: Lymph node metastasis
US: Ultrasonography
CT: Computed tomography
PET: Positron emission tomography
MRI: Magnetic resonance imaging
