# Supplementary figures and images for: Clinical study on postoperative recurrence in patients with pN0 esophageal squamous cell carcinoma
Source: J Cardiothorac Surg. 2014 Aug 28;9:150. doi: 10.1186/s13019-014-0150-4 (PMC4200175; doi:10.1186/s13019-014-0150-4)

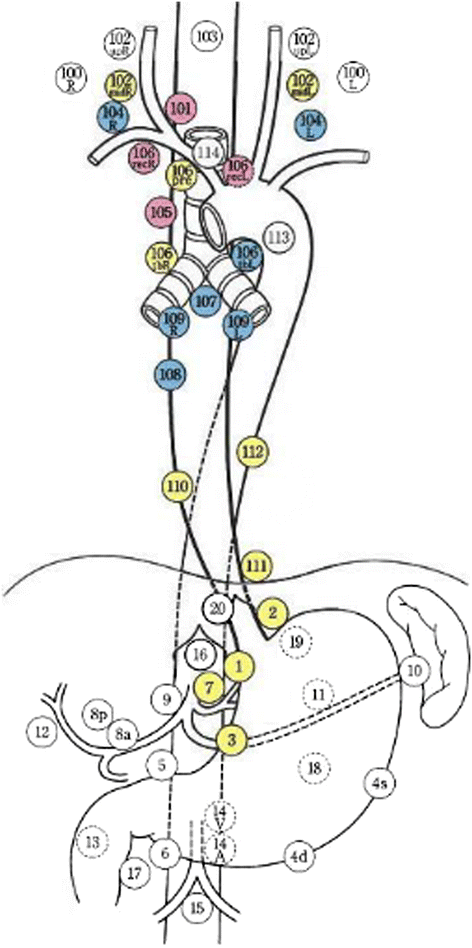

Supplement: Supplementary file 1 — Authors’ original file for figure 1 [file 13019_2014_150_MOESM1_ESM.gif]

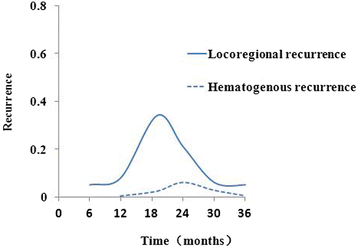

Supplement: Supplementary file 2 — Authors’ original file for figure 2 [file 13019_2014_150_MOESM2_ESM.gif]

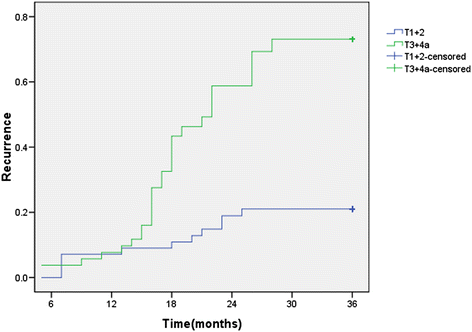

Supplement: Supplementary file 3 — Authors’ original file for figure 3 [file 13019_2014_150_MOESM3_ESM.gif]

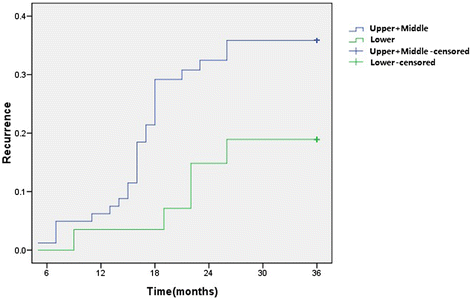

Supplement: Supplementary file 4 — Authors’ original file for figure 4 [file 13019_2014_150_MOESM4_ESM.gif]
